# Aberrant activation of five embryonic stem cell-specific genes robustly predicts a high risk of relapse in breast cancers

**DOI:** 10.1186/s12864-023-09571-3

**Published:** 2023-08-17

**Authors:** Emmanuelle Jacquet, Florent Chuffart, Anne-Laure Vitte, Eleni Nika, Mireille Mousseau, Saadi Khochbin, Sophie Rousseaux, Ekaterina Bourova-Flin

**Affiliations:** 1 Université Grenoble Alpes, INSERM U1209, CNRS UMR5309, EpiMed, Institute for Advanced Biosciences, Grenoble, France; 2 Université Grenoble Alpes, CHU Grenoble Alpes, Medical Oncology Unit, Cancer and Blood Diseases Department, Grenoble, France; 3 Université Grenoble Alpes, CHU Grenoble Alpes, Department of Pathology, Grenoble, France; 4 Université Grenoble Alpes, INSERM U1039, Bioclinical Radiopharmaceuticals, Grenoble, France

**Keywords:** Cancer/testis antigens, Breast cancer, Ectopic expression, Survival analysis, Prognosis biomarkers

## Abstract

**Background:**

In breast cancer, as in all cancers, genetic and epigenetic deregulations can result in out-of-context expressions of a set of normally silent tissue-specific genes. The activation of some of these genes in various cancers empowers tumours cells with new properties and drives enhanced proliferation and metastatic activity, leading to a poor survival prognosis.

**Results:**

In this work, we undertook an unprecedented systematic and unbiased analysis of out-of-context activations of a specific set of tissue-specific genes from testis, placenta and embryonic stem cells, not expressed in normal breast tissue as a source of novel prognostic biomarkers. To this end, we combined a strict machine learning framework of transcriptomic data analysis, and successfully created a new robust tool, validated in several independent datasets, which is able to identify patients with a high risk of relapse. This unbiased approach allowed us to identify a panel of five biomarkers, DNMT3B, EXO1, MCM10, CENPF and CENPE, that are robustly and significantly associated with disease-free survival prognosis in breast cancer. Based on these findings, we created a new Gene Expression Classifier (GEC) that stratifies patients. Additionally, thanks to the identified GEC, we were able to paint the specific molecular portraits of the particularly aggressive tumours, which show characteristics of male germ cells, with a particular metabolic gene signature, associated with an enrichment in pro-metastatic and pro-proliferation gene expression.

**Conclusions:**

The GEC classifier is able to reliably identify patients with a high risk of relapse at early stages of the disease. We especially recommend to use the GEC tool for patients with the luminal-A molecular subtype of breast cancer, generally considered of a favourable disease-free survival prognosis, to detect the fraction of patients undergoing a high risk of relapse.

**Supplementary Information:**

The online version contains supplementary material available at 10.1186/s12864-023-09571-3.

## Background

Breast cancer is the leading cancer in women in terms of incidence and mortality, with more than 2.1 million new cases reported and 627,000 deaths in 2018 worldwide. Although hereditary and genetic factors, such as a personal or family history of breast or ovarian cancer and inherited mutations in breast cancer susceptibility genes, including BRCA1 and BRCA2, account for 5% to 10% of breast cancer cases, nonhereditary factors remain major drivers of the observed world-wide and interethnic differences in incidence [1].

Breast cancer can be subdivided into different subgroups based on molecular and phenotypic characteristics, which are responsible for significant disparities in survival. Based on extensive transcriptomic analyses, at least four tumour subtypes have been described, which include luminal A, luminal B, human epidermal growth factor receptor 2 (HER2)-amplified and basal-like. This stratification of molecular subtypes in breast cancer extends the simpler histological classification based on immunohistochemical characteristics such as hormone receptor expression (oestrogen and progesterone receptors) and HER2 amplification. Genetic mutations, DNA methylation, copy number and protein expression complete this classification [2].

However, despite progress in research and therapy, breast cancer remains a world-wide public health issue. Early diagnosis, timely treatment and accurate prognosis evaluation are crucial determining factors for breast cancer survival rates. The search for reliable biomarkers still remains a challenge for biomedical scientists. Due to inherent tumour heterogeneity, in addition to the immunohistochemical and molecular subtypes, there is still a need for the identification of robust biomarkers that could accurately predict the behaviour of breast cancers, which would help the selection of appropriate treatment strategies. Additionally, the identification of candidate factors for targeted therapies would be of great help in the development of personalized treatments, which would efficiently and durably harness cancer cells, while minimizing the effects on healthy cells.

In all cancers, including breast cancer, genetic and epigenetic alterations result in aberrant gene expression with a significant contribution of normally silent tissue-specific genes [3]. Various investigations during the past decencies have revealed that a high proportion of all identified tumour-associated illegitimately activated genes in cancer corresponds to genes that are exclusively or predominantly expressed in male germ cells [4–8]. Moreover, the results of several studies suggest an association between the expression of these genes and poorer outcomes across a broad spectrum of solid tumours, as well as a higher prevalence in undifferentiated and advanced-stage cancers [9]. However, the out-of-context expression of tissue-specific genes is not restricted to testis-specific genes and many other genes with an exclusive or predominant pattern of expression in different tissues, such as placenta-specific genes, are also aberrantly activated in various cancers [3].

Molecular functional investigations of some the proteins encoded by a number of these tissue-specific genes clearly demonstrated that they contribute to the acquisition of new properties by cancer cells. For instance, data from our laboratory as well as from other groups indicated that the activation of the testis-specific protein NUT in the rare but highly aggressive NUT carcinoma completely modify CBP/p300-dependent chromatin acetylation signalling [10–13]. Illegitimate expression of the protein CYCLON in the diffuse large B-cell lymphomas controls cell response to Rituximab and tumour growth [14]. In addition, the aberrant expression of the mitochondrial protein FASTKD1 in aggressive forms of acute lymphoblastic leukaemia, impacts mitochondrial activity and drives an oncogenic epigenome reprogramming [15–17]. Finally, the out-of-context activity of *ATAD2*, a gene predominately expressed germline/embryonic stem cell, affects histone chaperone-bound chromatin dynamics and could promote oncogenic genome reprogramming [18–20]. Aberrant activation of a placenta-specific gene, ADAM12, favouring tumour invasion, activated following an oncogenic signalling pathway, is another example [21]. Additionally, there are accumulating data in the literature showing that some of the known “official” Cancer Testis genes (CTdatabase, http://www.cta.lncc.br) [22], also significantly contribute to malignant cell transformation [23–25]. Therefore, it is reasonable to hypothesize that out-of-context expression of tissue-specific genes could also play important roles in tumour progression and metastasis in most cancers, including breast cancer. Hence, their specific expression in cancer cells, and their lack of expression in the majority of non-germline cells also make the product of genes an attractive target for anti-cancer therapy.

Here we undertook a systematic investigation of ectopic activation of a set of genes specifically or predominantly expressed in testis, placenta and embryonic stem cells, based on an analysis of publicly available expression data from eight independent well-documented breast cancer cohorts. We first used transcriptomic data from normal human tissue samples to consider the normal expression profiles of all the annotated human genes, and to single out those that show a clear predominance of expression in a given tissue in an unbiased manner. An analysis of expression data using publicly available transcriptomes obtained from eight large cohorts of well-annotated breast tumours was then performed in order to identify among our germ cells, placenta, and ES cell—genes, the best candidates that could be proposed to design prognosis tests and/or to use as targets for future therapeutic developments. To this end, we established a method to reliably define ectopic tissue-specific gene expression in breast cancer cells, and then considered their association with survival on a ON (expressed) / OFF (not expressed) basis. Based on this approach, we show that a combination of several of these ectopically activated genes (ON genes) provides a powerful mean to detect the worse prognosis in breast cancers. These investigations not only highlighted a number of robust breast cancer biomarkers, but also helped to molecularly characterize the most aggressive fraction of breast tumours thanks to our ability to isolate these subpopulations of breast cancer.

## Results

### Many tissue-specific genes are frequently activated in breast cancer

Using the publicly available RNA-seq data of several normal tissues provided by GTEX and NCBI Sequence Read Archive, we computed the patterns of expression for all annotated genes. To identify genes with a tissue-predominant expression, we used an outlier detection technique based on the Z-score as presented in the Methods section. In total, 1882 tissue-specific genes encoding for testis, placenta or ESC were found. The patterns of expression of the 1882 selected genes in normal tissues are shown in Supp. Figure S1A. The tissues where each of these genes are predominantly expressed (testis, placenta, ESC) are listed in Supp. Table S1. A Venn diagram in Supp. Figure S1B presents the number of genes that show a shared tissue-specific expression pattern.

**Fig. 1:**
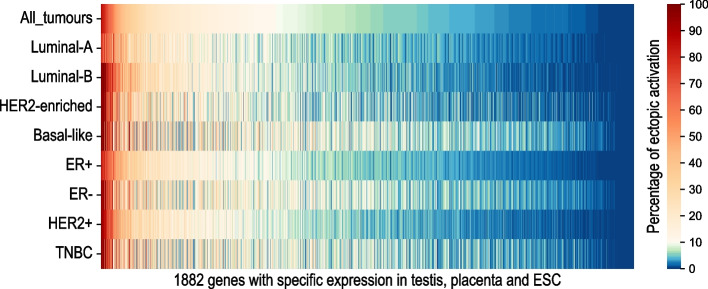
Heatmap showing the percentage of ectopic activations of the 1882 tissue-specific genes encoding for testis, placenta and embryonic stem cells in the total TCGA-BRCA dataset and in breast cancer subtypes. Frequent ectopic activations above the threshold of 10% are presented in red colour map. Infrequent ectopic activations below 10% are shown in blue colour map

For each of the 1882 tissue-specific genes, we used the transcriptomic data from breast non-tumour tissues (where these genes are silent) of the dataset TCGA-BRCA to establish a threshold of signal below which the gene was considered as not expressed. The threshold of expression was defined as the mean signal + two standard deviations calculated in the non-tumour breast samples. Based on this threshold value, for each gene, we measured the proportion of breast tumour samples where the gene was expressed (signal over the threshold, considered as ON). The results are shown as a heatmap in Fig. 1. These data confirm that, as expected, many tissue-specific genes are aberrantly expressed in a number of breast tumours, with variable frequencies, depending on the gene and the breast tumour molecular subtype. The frequencies of ectopic activations for each gene are presented in Supp. Table S2. Taking all subtypes together, 626 genes (33.2%) were found to be frequently activated, which by convention means, in more than 10% of breast cancer samples. This procedure allows us to remove the non-eligible genes with infrequent activations and consider only the genes that are ectopically expressed in a representative population of patients. This preliminary selection ensures their potential usability as biomarkers in routine clinical practice for all patients with breast cancer. Finally, we retained 626 frequently activated genes for the subsequent survival analysis.

However, this approach has a limitation in terms of the representation of epithelial cells in both non-tumour and tumour samples. In bulk RNA-seq data, the non-tumour breast tissues can potentially contain less epithelial cells and more stroma cells and adipocytes compared to the tumour samples. In this case, the expression level of the genes expressed in epithelial cells may be underestimated in the non-tumour samples due to their low representation. To accurately address this point, one would need to analyse single cell data which are unfortunately unavailable in the TCGA-BRCA cohort. For this reason, we selected the genes potentially activated in tumours in this preliminary step and then we relied on a thorough full machine learning approach described in next section to accurately identify the thresholds of gene activations during the survival analysis.

### A combination of five ectopically expressed genes robustly predicts patients’ prognosis in four independent test datasets

For each of the selected 626 tissue-specific genes, we performed a survival analysis as described in the Methods section. Briefly, we identified the genes for which it was possible to establish a stable activation threshold (defining the “OFF/ON” status of the corresponding gene), associated with patients’ survival prognosis, based on criteria explained below. This approach separated patients in two groups with significantly different survival probabilities. For this purpose, we used a specific dedicated approach based on machine learning principles to reduce possible artefacts and overfitting issues in survival analysis. The main steps of the method are summarized in Supp. Figure S2.

The idea behind this approach is that for some of these genes, their “ON” expression status may empower tumour cells with new properties of higher aggressiveness, resulting in a poor survival prognosis for patients. The main objective of our specific survival analysis approach, was to accurately identify thresholds of expression above which the activation of the genes was significantly and robustly associated with survival. It was also important to reject false positive results that could appear during the overall learning process, due to overfitting and possible heterogeneity among different datasets.

We considered that a threshold was stable when the following criteria were fulfilled: i/ a small modification of the threshold didn't impact significantly the prediction of the survival model; ii/ a random selection of subsets of samples produced the best similar results of the survival model; iii/ selected thresholds were validated across several datasets. These constraints were implemented through a formal machine learning pipeline separating all breast cancer cohorts into the training, validation and test datasets. In addition, we used a technique of random threefold cross-validations to introduce perturbations of the subsets of samples during the training step. The details are provided in the Methods section. The calculations were performed in several steps and described below.

First, in the training dataset TCGA-BRCA we identified 28 genes for which a stable threshold was detected and the activated status of the gene (ON) was significantly associated with a shorter disease-free survival probability (*p*-value < 0.05, FDR < 0.2). In the second step, we used three validation datasets GSE25066, GSE21653 and GSE42568 to include a possible heterogeneity across different datasets in the learning process and to evaluate the robustness of the selected genes. These 28 genes were then ordered according to the *p*-values obtained by the logrank tests in each validation dataset (Supp. Table S3).

These results showed that the gene DNMT3B was found significantly associated with disease-free survival in all three validation datasets (*p*-value < 0.05). Another four genes EXO1, MCM10, CENPF and CENPE were found significantly associated with disease-free survival in 2 of 3 validation datasets and in the third dataset the obtained *p*-value was also relatively low (*p*-value < 0.1). We selected these five genes as candidate biomarkers. For all other genes, we obtained high non-significant *p*-values in at least one dataset. These genes were considered not sufficiently robust; therefore, they were not validated. All five genes have normal predominant expression profile in embryonic stem cells; they are also expressed in testis at lower levels (Supp. Figure S3). The individual Kaplan–Meier survival curves for five selected genes in the training and validation datasets are shown in Supp. Figure S4.

Finally, the five candidate genes DNMT3B, EXO1, MCM10, CENPF and CENPE were combined in a new prognosis tool, Gene Expression Classifier or GEC, that stratifies patients according to the number of activated genes in the corresponding tumour (activation status ON). The patients for which none or only one gene is activated in the tumour have a more favourable disease-free survival prognosis than the patients for which two or more genes are activated. To ensure the robustness of our GEC tool, we tested it in four independent breast cancer cohorts E-MTAB-365, Miller-2005, Naderi-Caldas-2007 and Yau-2010 that had never been used either during the learning process nor for the selection of the five genes in the GEC.

**Fig. 2:**
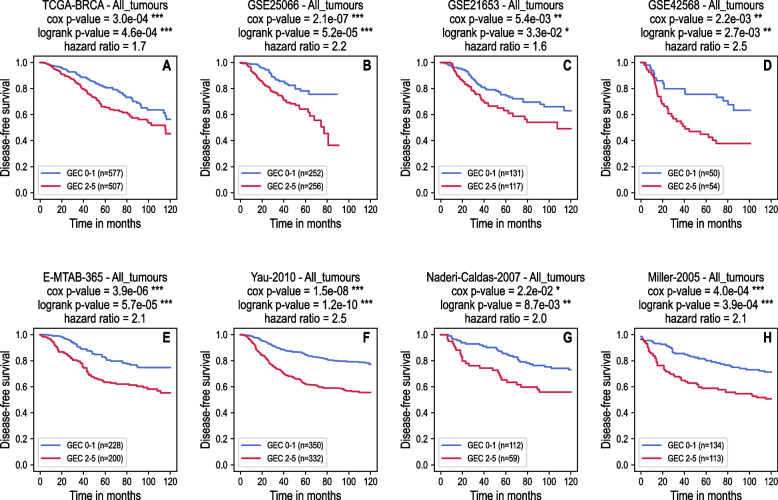
Kaplan–Meier survival curves showing disease-free survival probability according to the number of activated genes in the GEC tool for eight breast cancer datasets. **A**: Training dataset. **B-D**: Validation datasets. **E–H**: Test datasets. For each dataset, blue lines show the survival curves for the group of patients in which the corresponding tumours activated 0 or 1 gene in the GEC tool (GEC 0–1). Red lines represent the group of patients in which the tumours activated 2 or more genes (GEC 2–5). The *p*-values obtained from the logrank test and Cox proportional hazard model as well as the hazard ratios are displayed on the top of each plot. Significance symbols: * for *p*-value < 0.05, ** for *p*-value < 0.01, *** for *p*-value < 0.001

The results of the GEC performance in the test cohorts are shown in Fig. 2E-H. Our new GEC tool accurately predicted patients’ disease-free survival prognosis in all test datasets, providing significant *p*-values < 0.05 for the logrank test between two groups GEC 0–1 (favourable prognosis) and GEC 2–5 (unfavourable prognosis), as well as for Cox proportional hazard model considering the number of activated genes in the GEC as an explanatory variable. For information, we also presented the results of the GEC tool in the training dataset (Fig. 2A) and in the validation datasets (Fig. 2B-D) that were also significant, as expected.

### Multivariate analysis shows that the GEC tool provides complementary information to known risk factors

To evaluate the impact of the new GEC prognosis tool compared to other known risk factors in breast cancer, we performed a multivariate survival analysis using Cox proportional hazard model with the following explanatory variables: GEC, age, molecular subtype and tumour stage. For this analysis we selected the datasets with available annotations of the molecular subtype. Five datasets were eligible: TCGA-BRCA, GSE25066, GSE21653, E-MTAB-365 and Yau-2010. When the information about age or tumour stage was missing in some datasets, we used the available covariates. The results of the multivariate analysis are shown in Table 1.

**Table 1:** Results of multivariate survival analysis including our new GEC classifier and other known risk factors in breast cancer

	**TCGA-BRCA**		**GSE25066**		**GSE21653**		**E-MTAB-365**		**Yau-210**	
	*p-value*	*HR*	*p-value*	*HR*	*p-value*	*HR*	*p-value*	*HR*	*p-value*	*HR*
***GEC***	**0.011**	1.2	**0.005**	1.2	**0.01**	1.3	**0.039**	1.2	** < 0.001**	1.2
***Age***	** < 0.001**	1	0.786	1	0.749	1	0.685	1	NA	NA
***Molecular subtype***	0.591	1.1	0.059	1.2	0.314	0.9	0.054	1.3	0.933	1
***Stage***	** < 0.001**	2.3	NA	NA	NA	NA	0.466	1.2	NA	NA

The table shows *p*-values and hazard ratios (HR) obtained by multivariate Cox model for the covariates GEC, age, molecular subtype and tumour stage in five breast cancer datasets. Significant *p*-values < 0.05 are shown in bold. The symbol NA means that the corresponding explanatory variable was missing in clinical annotations of the dataset and was excluded from the analysis

In all considered datasets, the GEC tool provided significant and stable prediction of disease-free survival probability while corrected for other risk factors. It means that the GEC brings a new complementary information to known risk factors. On this basis, we conclude that it is potentially interesting to integrate the GEC classification to the existing breast cancer classifications criteria that are already using age, molecular subtype and tumour stage as explanatory variables to estimate patients’ survival prognosis.

### The GEC tool identifies patients with a high risk of relapse inside the luminal-A and luminal-B subtypes

**Fig. 3:**
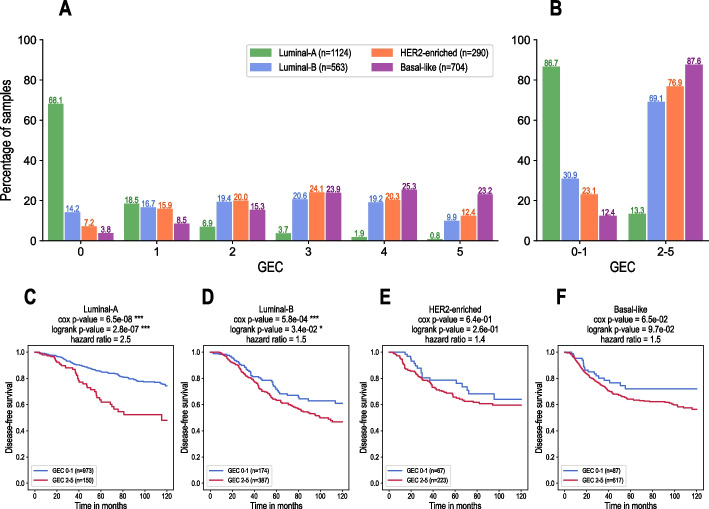
Results of the GEC tool in molecular subtypes of breast cancer. **A**: Distribution of breast cancer samples for five pooled datasets (TCGA-BRCA, GSE25066, GSE21653, E-MTAB-365 and Yau-2010) in luminal-A, luminal B, HER2-enriched and basal-like subtypes according to the number of activated genes in the GEC panel. The bar plots show the percentage of samples for each GEC group (from GEC 0 to GEC 5) in each molecular subtype. **B**: Same for the groups GEC 0–1 and GEC 2–5. **C-F**: Kaplan–Meier survival curves showing disease-free survival probability in luminal-A, luminal B, HER2-enriched and basal-like subtypes, respectively, according to the number of expressed genes in the GEC panel, presented in two groups: GEC 0–1 and GEC 2–5. The *p*-values obtained from the logrank test and Cox proportional hazard model as well as the hazard ratios are displayed on the top of each plot. Significance symbols: * for *p*-value < 0.05, ** for *p*-value < 0.01, *** for *p*-value < 0.001

In five datasets TCGA-BRCA, GSE25066, GSE21653, E-MTAB-365 and Yau-2010 we identified the GEC status for all samples and then performed survival analysis separately in each molecular subtype of breast cancer. Figure 3A-B show the distribution of all samples (five datasets pooled, *n* = 2681) in luminal-A, luminal B, HER2-enriched and basal-like subtypes according to the number of activated genes in the GEC panel.

We found that the majority of tumours in luminal-A molecular subtype, which is generally considered of a good prognosis, does not express any of the five genes of the GEC panel (68.1%) or only one gene of five (18.5%) as shown in Fig. 3A. However, 13.3% of tumours in this subtype still express between 2 and 5 GEC genes, corresponding to aggressive forms of breast cancer (Fig. 3B). Survival curves in Fig. 3C shows that this particular group of patients have a significantly higher risk of relapse compared to other patients with luminal-A subtype of breast cancer. Similarly, in luminal-B molecular subtype, the GEC tool is able to identify a subset of patients with a significantly higher risk of relapse (Fig. 3D). These results indicate that the GEC tool can be particularly interesting to use in clinical practice to efficiently identify the fraction of patients with a high risk of relapse inside the group of a priori favourable disease-free prognosis.

In HER2-enriched and basal-like subtypes, respectively, we didn't find significant association between the GEC status and disease-free survival (Fig. 3E-F). This result is probably due to an uneven distribution of the molecular subtypes in the training dataset, containing a majority of luminal A subtype (53.4%), whereas the proportion of the other molecular types was significantly lower (19.8% of luminal-B, 8.0% of HER2-enriched and 18.8% of basal-like). Therefore, since the majority of samples belongs to the luminal-A molecular subtype, the identification of the GEC biomarkers during the learning process was mostly impacted by this subtype. The HER2-enriched subtype was not sufficiently represented compared to the other subtypes. This could be the reason why we didn't find significant association with survival within this subtype. In basal-like subtype the obtained *p*-values were close to significant for both the logrank test and Cox model (cox *p*-value = 0.065, logrank *p*-value = 0.097, Fig. 3F), suggesting that the patients in the group basal-like and GEC 0–1 may have a tendency for a lower risk of relapse.

### Gene Set Enrichment Analysis (GSEA) shows shared molecular signatures of the aggressive GEC + tumours in several breast cancer datasets

**Fig. 4:**
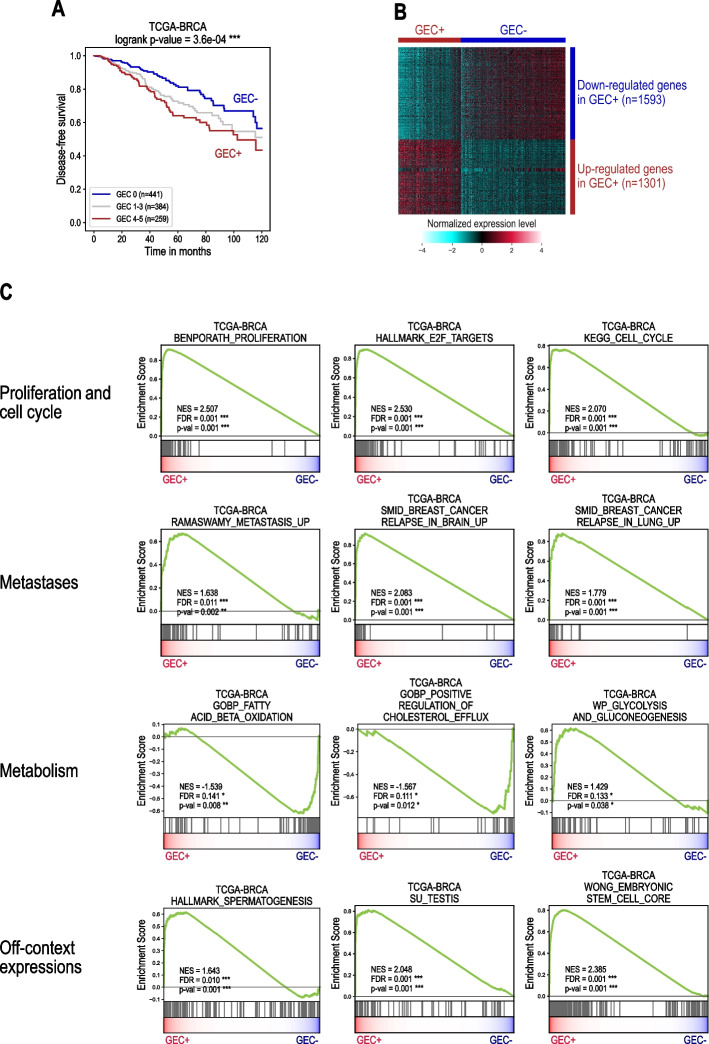
Main results of the GSEA analysis for transcriptomic profiles of GEC + versus GEC- tumours in the dataset TCGA-BRCA. **A**: Kaplan–Meier disease-free survival curves between the group of tumours without GEC ectopic expressions (GEC-) and those with major GEC ectopic expressions of 4 or 5 genes (GEC +). The displayed *p*-value corresponds to the logrank test between GEC- and GEC + groups. **B**: Heatmap of the differential expression profiles of GEC + versus GEC- in TCGA-BRCA. The differentially expressed genes used for the heatmap were selected with an adjusted *p*-value < 0.05 of Mann–Whitney test and abs (ratio) > 1.5. The hierarchical clustering was performed using Euclidian-based distance with Ward’s linkage for samples and Pearson correlation for genes. **C**: GSEA plots illustrating main enrichment/depletion profiles in GEC + tumours compared to GEC- tumours in the dataset TCGA-BRCA. For all the gene sets, the enrichment or depletion was considered significant with a nominal *p*-value < 0.05 and FDR < 0.25. The gene sets were selected from the MSigDB database of the Broad Institute (collections C2, C5 or H of the MsigDB)

Differential expression analysis and the corresponding GSEA were performed in ten independent breast cancer datasets (see Supp. Table S4) between the group of tumours without GEC ectopic expressions (GEC-) and those with major GEC ectopic expressions of 4 or 5 genes (GEC +). The intermediate group of tumours classified in GEC 1–3 was excluded from the differential analysis. We considered here only the extreme GEC groups in order to identify the most striking differences in molecular signatures between the aggressive tumours GEC + and the tumours of favourable prognosis GEC-. The survival curves of the groups GEC- and GEC + in the dataset TCGA-BRCA are shown in Fig. 4A as an example. The heatmap in Fig. 4B illustrates the expression of the genes down- and up-regulated in GEC + versus GEC- breast cancer samples with an absolute fold change of expression values above 1.5 and an adjusted Mann–Whitney *p*-value < 0.05. Respectively, 1593 and 1301 genes were down- and up- regulated in the dataset TCGA-BRCA.

**Fig. 5:**
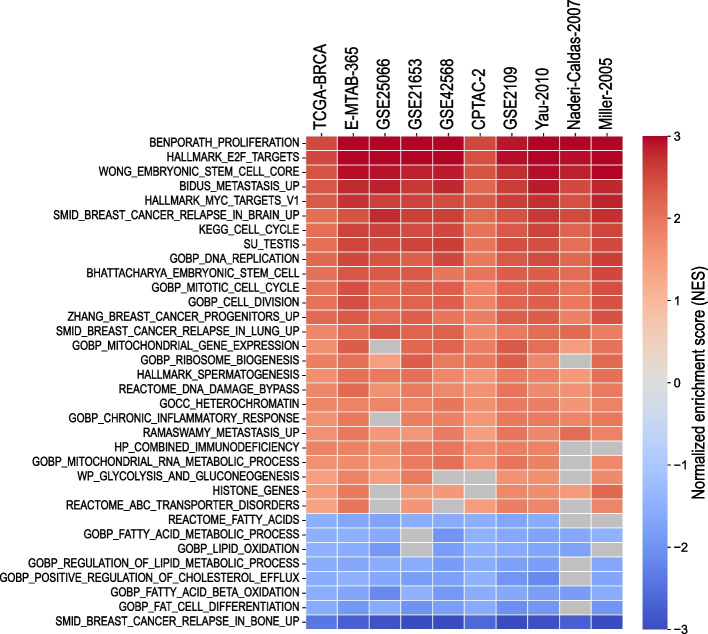
Gene Set Enrichment Analysis (GSEA) shows consistent molecular signatures of the aggressive GEC + tumours in several breast cancer datasets. The heatmap represents the normalized enrichment score (NES) obtained from the GSEA analysis in ten breast cancer datasets for different genes sets. Significantly enriched gene sets are shown in red colours; significantly depleted gene sets are displayed in blue colours. For all the gene sets, the enrichment or depletion was considered significant with a nominal *p*-value < 0.05 and FDR < 0.25. Grey cells correspond to non-significant results

In order to characterize the molecular profile of GEC + aggressive breast tumours, we performed Gene Set Enrichment Analysis (GSEA) for all 10 datasets to highlight biological pathways correlating with GEC + compared to GEC- samples (Figs. 4C and 5). The GSEA profiles of the aggressive GEC + form of breast cancer revealed a significant and consistent up-regulation in gene sets involved in cell proliferation and cell cycle progression. In addition, the GEC + tumours were found significantly enriched in the signatures of metastatic breast cancers prone to develop distant metastases in brain and lung. Interestingly, many pathways related to cholesterol and fatty acid metabolism were significantly depleted in the majority of breast cancer datasets in the GEC+ tumour fraction; however, the mitochondrial gene expression and mitochondrial RNA metabolic processes did not seem to be directly affected. Finally, the GEC + tumours were found significantly enriched in the gene sets of spermatogenesis, testis and embryonic stem cells in all datasets, indicating massive ectopic activations of these genes in aggressive forms of breast cancers.

## Discussion

Several cancer testis antigens present in the “official” list of CTAs were proposed in the literature as potential diagnostic or prognostic biomarkers in breast cancer. These signatures, however, were not sufficiently validated in independent studies, producing sometimes controversial results [26], and, therefore, cannot be applied in clinical practice. Nowadays, an important number of transcriptomic breast cancer datasets are available in public data repositories, making possible a thorough full machine learning approach for biomarker discovery with extensive validations and tests in various breast cancer cohorts.

Using a large dataset of RNA-seq data in normal tissues from GTEX and NCBI repositories, as well as eight independent breast cancer cohorts, we applied a strict machine learning pipeline in order to accurately define all genes with predominant expression profiles in testis, placenta or ESC, and to identify among these genes the most robust biomarkers to predict disease-free survival prognosis. These analyses revealed five genes, DNMT3B, EXO1, MCM10, CENPF and CENPE, that are normally not expressed in healthy breast tissue but become frequently activated in breast cancers. In addition, the aberrant activation of these genes was found systematically associated with a shorter disease-free survival in several cohorts.

On the basis of these findings, we combined the five biomarkers to create a new prognosis tool Gene Expression Classifier (GEC) that stratifies patients according to the number of ectopically activated genes in the GEC panel. A higher number of aberrant activations of these genes significantly correlated with a shorter disease-free survival prognosis of patients in all eight datasets. In particular, we proposed to stratify patients to the group of favourable survival prognosis if the number of ectopically activated genes found in the tumours was equal to 0 or 1 (GEC 0–1) and to the group of unfavourable survival prognosis if the number of ectopically activated was higher (GEC 2–5).

A multivariate survival analysis in five independent datasets demonstrated that the GEC tool remained significantly predictive after the adjustment for other risk factors as molecular subtype, patient age and tumour stage, also related to prognosis. We also found that the GEC tool was particularly efficient to detect tumours with a high risk of relapse inside the molecular subtypes luminal-A and luminal-B.

The five biomarkers identified in this work, DNMT3B, EXO1, MCM10, CENPF and CENPE, have predominant expression profiles in embryonic stem cells and are also expressed in testis. The literature may highlight potential mechanisms which could be involved in the oncogenic activities of five genes and explain the strong association between their expression and aggressive forms of breast cancer.

DNMT3B is a DNA methyltransferase that regulates the epigenome by de novo methylation of CpG sites. Aberrant activation of DNMT3B in breast cancer was reported in [27]. Recently, it was also shown that DNMT3B is induced in metastatic cells and facilitates distant colonization [28] and that high DNMT3B levels are correlated with poor patient survival and more aggressive subtypes of breast cancer [29]. Preclinical studies demonstrated that overexpression of DNMT3B promotes primary tumour progression in melanoma and colon cancer [30, 31].

EXO1 encodes a protein with 5' to 3' exonuclease activity as well as an RNase H activity. It is implicated in several genomic DNA metabolic processes such as replication stress response, double strand break repair, mismatch repair, nucleotide excision repair and telomere maintenance. However, even though EXO1 is of paramount importance to generate signals for the proper DNA damage response, Sertic and colleagues [32] argued that an overexpression of EXO1 can results in excessive nucleolytic activity, which leads to increased genome instability and alterations in cellular functions. Interestingly, in agreement with our data, an overexpression of EXO1 has been already reported to be associated with poor prognosis in breast and lung cancers [33, 34].

The protein encoded by the gene MCM10 is one of the highly conserved mini-chromosome maintenance proteins family (MCM) that are involved in the initiation of eukaryotic genome replication. Mughal and colleagues [35] observed that MCM10 promotes tumorigenic properties in immortal non-tumorigenic mammary cells by increasing proliferation, shortening the cell cycle, and promoting tumorigenic characters in *in-vivo* mimicking conditions. MCM10 was also suggested as a potential prognostic biomarker in breast cancer [36] and in hepatocellular carcinoma [37].

The genes CENPE and CENPF encode proteins associated with the centromere-kinetochore complex. CENPF associates with the kinetochore and maintains this association through early anaphase. The localization of this protein suggests that it may play a role in chromosome segregation during mitosis. CENPE is not present during interphase and first appears at the centromere region of chromosomes during prometaphase. This protein is required for stable spindle microtubule capture at kinetochores, which is a necessary step in chromosome alignment during prometaphase. High expression of both CENPE and CENPF was associated with low oestrogen and progesterone receptor expression levels in breast cancer [38]. The expression of these genes was also reported to be associated with progression and unfavourable prognosis in retinoblastoma, oesophageal adenocarcinoma, melanoma and hepatocellular carcinoma [39–42].

Although some of these genes have already been described in the literature as prognostic factors in breast cancer, our work provided a systematic and comprehensive exploration of known and unknown candidate prognosis biomarkers. An important input of this work is the evaluation of the prognosis values of these selected markers compared to many others reported in the literature, that were not validated following our rigorous systematic consideration. Indeed, as expected, within the list of our testis-specific genes there were many of the known “official” cancer-testis antigens from CTdatabase [22]. However, none of them were found on our GEC.

Interestingly, the GSEA analysis revealed a massive overexpression of testis-specific, ESC-like specific genes as well as the genes related to spermatogenesis in the GEC + aggressive forms of breast cancers, in total accordance with our hypotheses. However, more research is still required to characterize and understand the mechanisms involved in their oncogenic activities. Indeed, the role of these different genes in the tumorigenesis of breast cancer seems to be multiple, and involves different molecular mechanisms, some of which are still in need to be investigated. Additionally, our analysis also suggests that in many cases of breast cancer, several of these genes could be co-expressed. This co-expression suggests that they could contribute together to known or yet unknown oncogenic pathways, which remain to be investigated.

## Conclusions

This work highlights a subset of five tissue-specific genes whose expression is strongly and robustly associated with patients' survival. Our results are particularly encouraging to predict individual survival prognosis in breast cancer for each patient, especially at early stages of the disease in order to adapt the treatment. This approach can be potentially implemented not only with RNA-sequencing technique but also with RT-qPCR or immunohistochemistry tests which are usually more convenient and cost-effective in clinical practice. Such tests have been successfully developed for other cancer types in our previous studies, for example, in the case of oral squamous cell carcinoma [43] or T-cell acute lymphoblastic leukaemia [44]. Indeed, these five genes or their encoded proteins could be used by the scientific and medical communities as a basis for further mechanistic investigations of aggressive breast cancer as well as for the development of diagnostic/prognostic tools and the design of new targeted therapies.

## Methods

### Transcriptomic data

To obtain expression profiles in normal tissues, we used RNA sequencing (RNA-seq) data provided by the GTEX portal and NCBI Sequence Read Archive (datasets PRJNA280600, PRJEB4337, PRJEB2445, PRJNA270632, GSE70741, GSE53096). We also used 10 breast cancer datasets from public data repositories: GDC Data Portal, ArrayExpress, NCBI GEO and USCS Xena. The detailed description of the datasets is presented in Supp. Table S4.

The RNA-seq data of normal human tissues from GTEX repository and NCBI Sequence Read Archive contain 2955 samples of 48 different tissues: 2913 samples of 39 adult tissues, 37 samples of 8 foetal tissues and 5 samples of embryonic stem cells. Some tissues were pooled in more general tissue groups. In total, we obtained 26 tissue groups: 18 tissue groups for adult tissues, 7 tissue groups for foetal tissues and 1 tissue group for embryonic stem cells. The list of available normal tissues, tissues groups and the corresponding sample sizes is provided in Supp. Table S5.

The transcriptomic data of microarray datasets E-MTAB-365, GSE25066, GSE21653, GSE42568, GSE2109, Miller-2005, Naderi-Caldas-2007 and Yau-2010 were obtained with Affymetrix Human Genome Arrays U133 Plus 2.0, U133A and U133B. The data were normalized using Robust Multi-array Average (RMA) method [45] and then log-transformed. For the TCGA-BRCA and CPTAC-2 datasets, we used the RNA-seq values normalized by FPKM method directly provided by the GDC Data Portal. The FRKM values were log-transformed by taking log2(1 + FPKM). For the RNA-seq datasets of normal tissues, we downloaded pre-processed raw counts and normalized them in log-transformed RPKM units.

### Identification of genes with predominant expression profiles

To establish the expression profile of the genes in normal tissues, we used RNA-seq data from GTEX public repository and NCBI Sequence Read Archive (2955 samples, 48 tissues). We classified all the genes available in this dataset in two groups according to their expression profiles in normal tissues: predominant expression or ubiquitous expression. A predominant expression profile is defined as an expression pattern with one tissue determined as outlier in the distribution of expression values through tissues. These genes show no expression or lower expression levels in other tissues. To detect the predominant tissues, for each gene, we used an outlier detection technique based on the Z-score:

$$Z\ score=\frac{x-mean}{std}$$

where *x* corresponds to the average expression level in a given tissue.

The Z-score was calculated by subtracting the mean from the average expression levels in all tissues excluding foetal tissues, and then dividing the difference by the standard deviation. If the Z-score was found above a certain threshold, corresponding to 60% of the maximum Z-score [46] accordingly to the formula:

$$Z\ score\ threshold=0.6 \frac{ (N-1)}{\sqrt{N}}$$

where *N* is the total number of tissues, we considered the gene to be predominantly expressed in this tissue. The predominance of expression was analysed for each gene in both the detailed list of tissues (*N* = 40) and tissues groups (*N* = 19) with the corresponding Z-score thresholds 3.7 and 2.5, respectively.

We then selected the predominant genes in testis, placenta and embryonic stem cells that were not expressed or lowly expressed in normal breast (expression level in normal breast 10 times lower than in the predominant tissue). Applying these criteria, we identified a total of 1882 predominant genes listed in Supp. Table S1.

### Calculating the frequency of abnormal expression in breast cancer

Considering the 1882 predominant genes, we calculated the frequency of aberrant expression of these genes in breast cancer in the TCGA-BRCA dataset, where sufficient numbers of non-tumour and tumour samples were available. We set a threshold of expression to the mean + 2 standard deviations of the expression signal detected in non-tumour breast samples and then calculated the percentage of tumour samples in which the expression level was above the threshold.

### Survival analysis

We performed a dedicated survival analysis to explore the association between aberrant gene expression and disease-free survival and to identify robust prognostic biomarkers in breast cancer. Our biomarker discovery method is based on the published works of [3, 43]. In this study, we updated the original method by adding a machine learning framework in order to insure the reproducibility of the results in different breast cancer cohorts. The main steps of the pipeline are described below and also presented in Supp. Figure S2.

Eight cohorts with available survival data were separated in the training, validation and test datasets. The TCGA-BRCA cohort with the highest sample size and a long-term follow-up was designed as the training dataset. Three other cohorts GSE25066, GSE21653 and GSE42568 were used as the validation datasets. The objective of the validation step was to present to the algorithm the most heterogeneous cohorts in terms of sample composition, sample size and technology to retain the most stable biomarkers during the learning process. The additional four cohorts E-MTAB-365, Miller-2005, Naderi-Caldas-2007 and Yau-2010 were used as the test datasets to confirm our prognosis prediction tool in completely independent cohorts, never seen during the learning process.

In the training step, we checked if it was possible to define thresholds that could stratify patients into two groups with significantly different prognosis. With this purpose, for each gene in the TCGA-BRCA cohort, we tested all possible thresholds in the range from the 15th to the 85th percentile of expression in tumour samples, with a step of a half of percentile. All thresholds were analysed using logrank statistical test between the ON and OFF groups. We performed these tests in the total dataset as well as in the random subsets of samples generated within threefold cross-validations repeated five times. The obtained *p*-values were adjusted by Benjamini–Hochberg procedure. A threshold was considered as significant if the corresponding logrank *p*-value < 0.05, FDR < 0.2 and hazard ratio > 1. When several significant thresholds were present, we selected one reference threshold corresponding to the most stable threshold in all cross-validations.

In the validation step, we selected the genes for which at least one significant threshold associated with patients’ survival probability was found during the training step. For these significant thresholds, we determined their corresponding percentile ranking in the total distribution of tumour samples. We then propagated the thresholds, expressed as percentile rankings, to all other datasets. Subsequently, we performed the logrank test for these genes in the validation cohorts using the same threshold. The genes were ordered according to the obtained *p*-values and hazard ratios in all validation cohorts. Genes that achieved simultaneous significance in at least two out of the three validation cohorts, for which the *p*-values obtained in the third cohort were also relatively low < 0.1, were chosen as candidate biomarkers.

In the last step, the candidate biomarkers were combined to create a prognosis tool, named Gene Expression Classifier or GEC, which stratifies patients according to the number of aberrantly activated genes among these biomarkers. Finally, the combined GEC tool was tested in the independent test cohorts using the logrank test and Cox proportional hazard model.

The proposed dedicated approach (named “ectopy”) for systematic discovery of prognosis biomarkers in cancers from omics data was implemented in Python programming language. The code of “ectopy” tool is publicly available on Github repository https://github.com/epimed/ectopy.

### Gene Set Enrichment Analysis (GSEA)

The GSEA [47, 48] was carried out on the collections C2, C5 and H of gene sets made available by the Broad Institute in the database MSigDB (https://www.gsea-msigdb.org/gsea), using the GSEA software available on the website.

### Supplementary Information

**Additional file 1:** **Fig. S1.** (A) Heatmap showing the expression of 1882 genes in normal adult tissues with predominant expression in testis (male germinal), embryonic stem cells (ES cells) or placenta, and not expressed in normal breast (female genital). The expression levels of all genes are normalized by scaling each feature to a range between zero and one. The genes are ordered according their normalized expression levels in the tissues of interest (testis, placenta and ES cells, respectively). (B) Venn diagram showing the distribution of 1882 genes according the tissue of predominance: testis, embryonic stem cells and/or placenta. **Fig. S2. **Flow chart representing the main steps of the biomarker discovery pipeline. **Fig. S3. **Expression profiles in normal tissues of the five genes in the GEC panel DNMT3B, EXO1, MCM10, CENPF and CENPE based on RNA-seq data from GTEX and NCBI Sequence Read Archive. All five genes have a predominant expression profile in embryonic stem cells. They are also expressed in testis (male germinal) at lower levels. These genes are not expressed in normal breast and female genital tissues. **Fig. S4. **Kaplan-Meier individual survival curves of the genes DNMT3B, EXO1, MCM10, CENPF and CENPE in the training (TCGA-BRCA) and validation (GSE25066, GSE21653, GSE42568) datasets.

**Additional file 2:** **Table S1.** List of genes with predominant expression in testis, placenta and/or embryonic stem cells. **Table S2.** Frequencies of ectopic activations of the tissue-specific genes. **Table S3.** Results of the validation step in the biomarker discovery pipeline. **Table S4.** Datasets of normal tissues and breast cancers with corresponding sample sizes. **Table S5.** List of normal tissues and the corresponding sample sizes.

## Data Availability

The datasets analysed during the current study are publicly available (the corresponding identifiers are given in Supp. Table S4). The analysis pipelines and statistics are described in the Methods section. The Python code of “ectopy” tool implementing our approach for discovery of prognostic biomarkers from omics data is publicly available on Github repository https://github.com/epimed/ectopy. The full data outputs supporting the conclusions of this article are included within the article and its additional files.

## Abbreviations

CTA: Cancer Testis Antigens (CTAs)
ER: Oestrogen Receptor
ESC: Embryonic Stem Cell
FPKM: Fragments Per Kilobase of transcript per Million mapped reads
GDC: Genomic Data Commons Data Portal
GEC: Gene Expression Classifier
GEO: Gene Expression Omnibus
GTEX: Genotype-Tissue Expression (GTEx)
HER2: Human epidermal growth factor receptor 2
NCBI: National Center for Biotechnology Information
RMA: Robust Multi-array Average
RNA-Seq: RNA Sequencing
RPKM: Reads Per Kilobase Million
TAA: Tumour-associated antigens (TAAs)
TCGA: The Cancer Genome Atlas
TNBC: Triple Negative Breast Cancer
USCS: University of California Santa Cruz
