# Supplementary material for: Aberrant activation of five embryonic stem cell-specific genes robustly predicts a high risk of relapse in breast cancers
Source: BMC Genomics. 2023 Aug 17;24:463. doi: 10.1186/s12864-023-09571-3 (PMC10436393; doi:10.1186/s12864-023-09571-3)
Supplement: Supplementary file 1 — Additional file 1: Fig. S1. (A) Heatmap showing the expression of 1882 genes in normal adult tissues with predominant expression in testis (male germinal), embryonic stem cells (ES cells) or placenta, and not expressed in normal breast (female genital). The expression levels of all genes are normalized by scaling each feature to a range between zero and one. The genes are ordered according their normalized expression levels in the tissues of interest (testis, placenta and ES cells, respectively). (B) Venn diagram showing the distribution of 1882 genes according the tissue of predominance: testis, embryonic stem cells and/or placenta. Fig. S2. Flow chart representing the main steps of the biomarker discovery pipeline. Fig. S3. Expression profiles in normal tissues of the five genes in the GEC panel DNMT3B, EXO1, MCM10, CENPF and CENPE based on RNA-seq data from GTEX and NCBI Sequence Read Archive. All five genes have a predominant expression profile in embryonic stem cells. They are also expressed in testis (male germinal) at lower levels. These genes are not expressed in normal breast and female genital tissues. Fig. S4. Kaplan-Meier individual survival curves of the genes DNMT3B, EXO1, MCM10, CENPF and CENPE in the training (TCGA-BRCA) and validation (GSE25066, GSE21653, GSE42568) datasets. [file 12864_2023_9571_MOESM1_ESM.pdf]

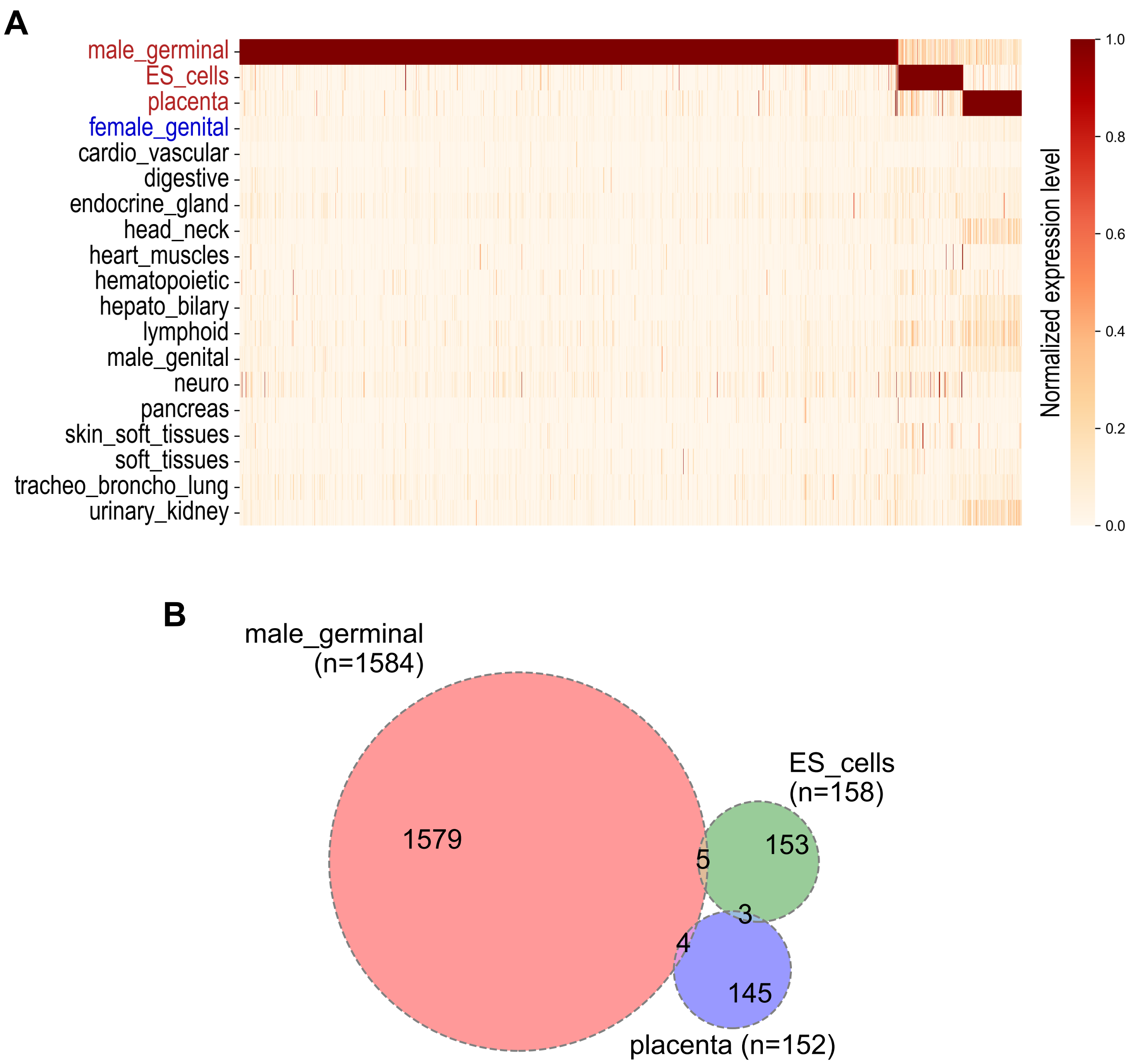

Fig. S1. (A) Heatmap showing the expression of 1882 genes in normal adult tissues with predominant expression in testis (male germinal), embryonic stem cells (ES cells) or placenta, and not expressed in normal breast (female genital). The expression levels of all genes are normalized by scaling each feature to a range between zero and one. The genes are ordered according their normalized expression levels in the tissues of interest (testis, placenta and ES cells, respectively). **(B)** Venn diagram showing the distribution of 1882 genes according the tissue of predominance: testis, embryonic stem cells and/or placenta.

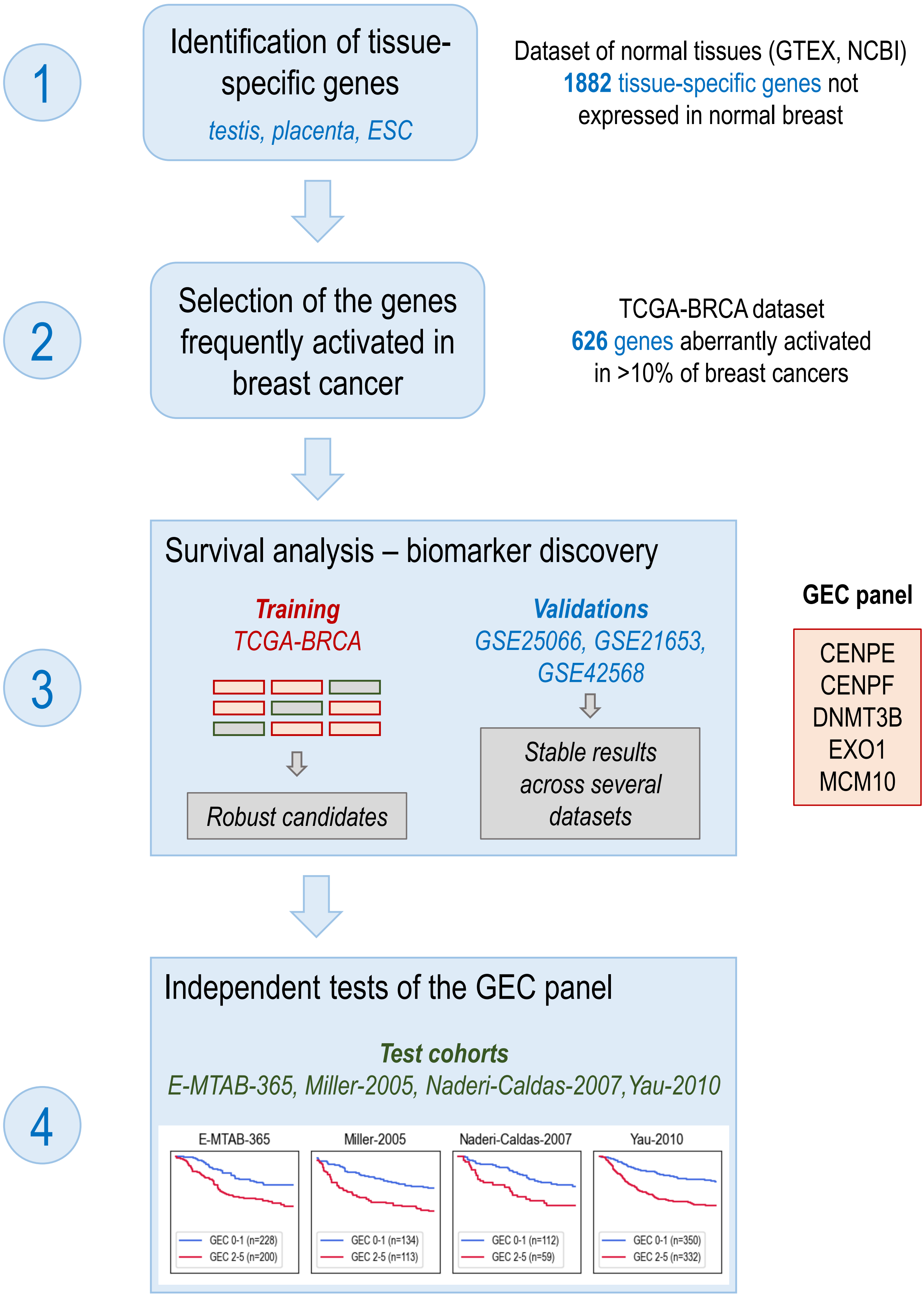

Fig. S2. Flow chart representing the main steps of the biomarker discovery pipeline.

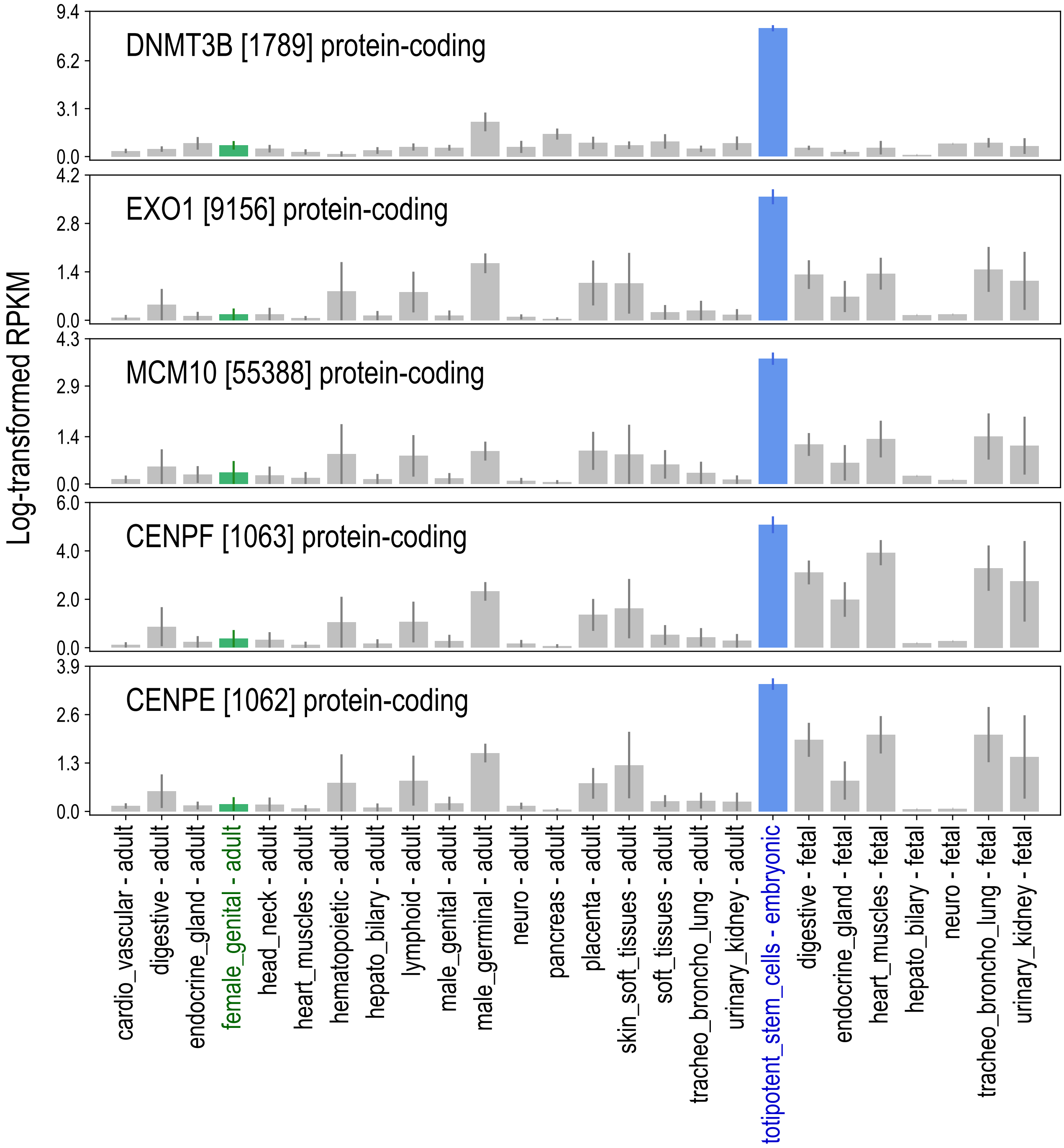

Fig. S3. Expression profiles in normal tissues of the five genes in the GEC panel DNMT3B, EXO1, MCM10, CENPF and CENPE based on RNA-seq data from GTEX and NCBI Sequence Read Archive. All five genes have a predominant expression profile in embryonic stem cells. They are also expressed in testis (male germinal) at lower levels. These genes are not expressed in normal breast and female genital tissues.

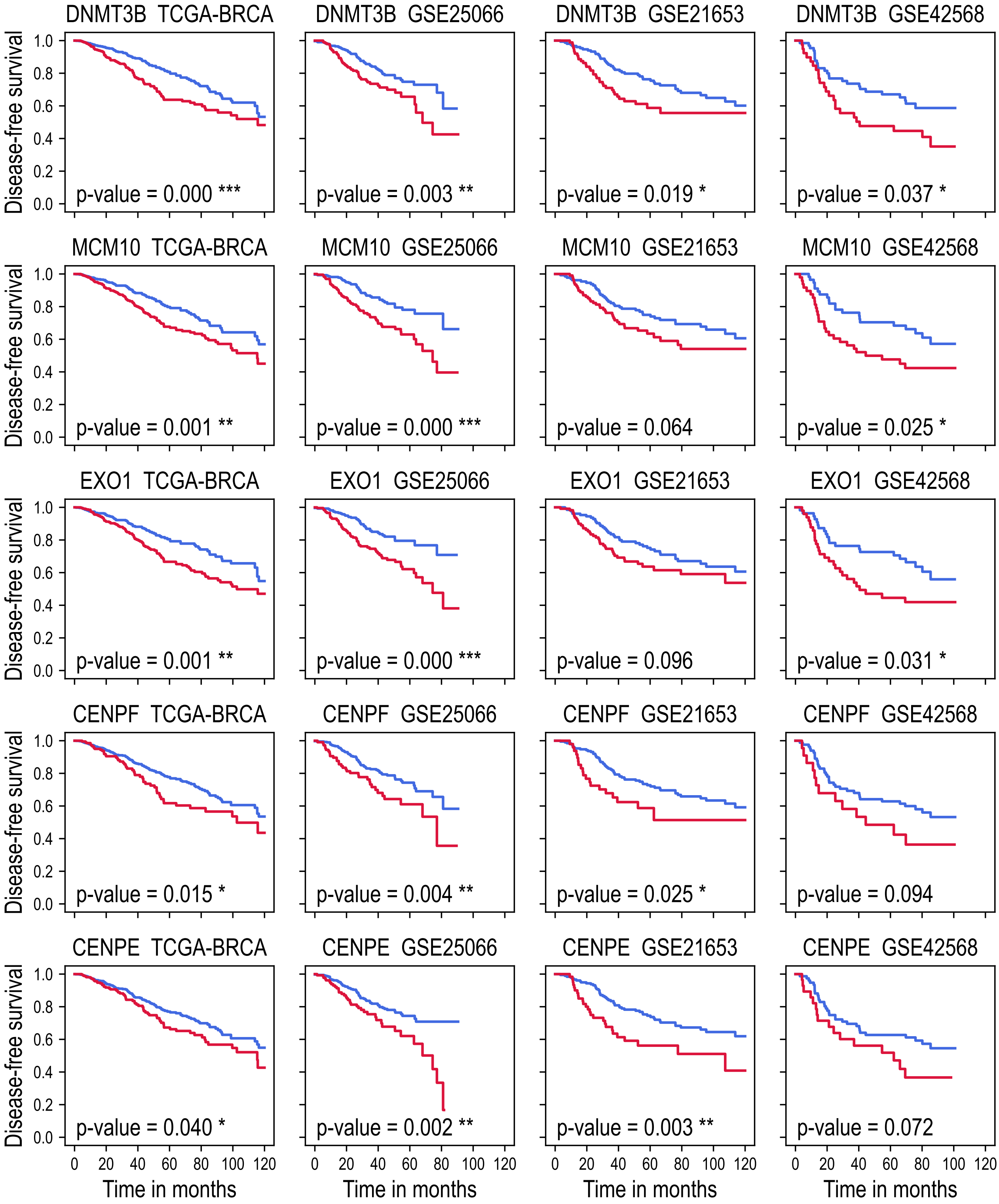

Fig. S4. Kaplan-Meier individual survival curves of the genes DNMT3B, EXO1, MCM10, CENPF and CENPE in the training (TCGA-BRCA) and validation (GSE25066, GSE21653, GSE42568) datasets.
